# Actions to support local charitable organizations in countering the spread of food insecurity

**DOI:** 10.1093/eurpub/ckac130.107

**Published:** 2022-10-25

**Authors:** G Barocco, E Croci, T Longo, A Calabretti, P Bogoni, J Sepe, A Marsi

**Affiliations:** 1 Servizio Igiene Alimenti e Nutrizione, Azienda Sanitaria Universitaria Giuliano Isontina, Trieste, Italy; 2 Department of Chemical and Pharmaceutical, Trieste University, Trieste, Italy; 3 Department of Economics, Business, Mathematics and Statistics, Trieste University, Trieste, Italy

## Abstract

**Background:**

According to the Italian Statistic Institute data, in Italy between 2007 - 2020 individual absolute poverty (AP) grew from 4.1 to 9.4%. In 2020 13.5% of minors lived in AP, furthermore in 2017 22.3% of Italians lived in conditions of food poverty or food insecurity (FI), between 2018 - 2019 1 in 7 minors lived in conditions of FI. The scientific literature underlines how inadequate food access is one of the risk factors for the onset of chronic non-communicable diseases. The aims of the project were to evaluate in the 3 main local charitable organizations (LCOs) the qualitative - quantitative profile of the food basket and the content of protective components present in fruits and vegetables (FV) available for socially disadvantaged communities (SDCs); determine the main nutritional critical points (NCPs); and identify possible corrective actions (CAs).

**Methods:**

The average composition of the food baskets available in 2021 for 1600 users by the 3 LCOs was compared with national nutritional recommendations. In February 2022, 23 samples of FV were collected from the LCOs for chemical analyses by the University of Trieste (UT). The outcomes were compared with the food composition database of the UT.

**Results:**

The food baskets at the LCOs were characterized by: daily average availability of 50 to 145 g of FV (8 to 22% of the recommended requirement); weekly average availability from 26 to 200g of cheeses, from 0 to 132g of meat. The quantitative profile of the protective molecules present in FV samples was reduced by 11 to 40 % compared to the UT database. The main NCPs were lack of nutrition guidelines on targeted food programmes and of monitor systems to evaluate the food basket balance for SDCs. CAs have been planned to integrate food safety and food security in public health programs to support LCOs.

**Conclusions:**

The results of the project can significantly support LCOs towards integrated actions in the food and nutritional policies at local and regional level.

**Key messages:**

- The rapid spread of food insecurity among the population, especially in families with minors, requires new tools to plan rapid and effective actions of food and nutrition policies.
- The analysis of nutritional critical points in the flux of donating food to socially disadvantaged communities is crucial for identifying corrective actions and to support charitable organizations.

